# A cross-sectional study of patients with and without substance use disorders in Community Mental Health Centres

**DOI:** 10.1186/1471-244X-11-93

**Published:** 2011-05-23

**Authors:** Linda E Wüsthoff, Helge Waal, Torleif Ruud, Rolf W Gråwe

**Affiliations:** 1 Norwegian Centre for Addiction Research, Institute of Clinical Medicine, University of Oslo, Oslo, Norway; 2 Department of Research and Development at the Division Mental Health Services, Akershus University Hospital, Lørenskog, Norway; 3 Institute of Clinical Medicine, University of Oslo, Oslo, Norway; 4 Department of Research and Development at the Alcohol and Drug Treatment Health Trust in Central Norway, Trondheim, Norway

## Abstract

**Background:**

Epidemiological studies have consistently established high comorbidity between psychiatric disorders and substance use disorders (SUD). This comorbidity is even more prominent when psychiatric populations are studied. Previous studies have focused on inpatient populations dominated by psychotic disorders, whereas this paper presents findings on patients in Community Mental Health Centres (CMHCs) where affective and anxiety disorders are most prominent. The purpose of this study is to compare patients in CMHCs with and without SUD in regard to differences in socio-demographic characteristics, level of morbidity, prevalence of different diagnostic categories, health services provided and the level of improvement in psychiatric symptoms.

**Methods:**

As part of the evaluation of the National Plan for Mental Health, all patients seen in eight CMHCs during a 4-week period in 2007 were studied (n = 2154). The CMHCs were located in rural and urban areas of Norway. The patients were diagnosed according to the ICD-10 diagnoses and assessed with the Health of the Nation Outcome Scales, the Alcohol Use Scale and the Drug Use Scale.

**Results:**

Patients with SUD in CMHCs are more frequently male, single and living alone, have more severe morbidity, less anxiety and mood disorders, less outpatient treatment and less improvement in regard to recovery from psychological symptoms compared to patients with no SUD.

**Conclusion:**

CMHCs need to implement systematic screening and diagnostic procedures in order to detect the special needs of these patients and improve their treatment.

## Background

Epidemiological studies have consistently established high comorbidity between psychiatric disorders and substance use disorders (SUD) [1-5]. By SUD we refer to abuse, dependence and addiction from both alcohol and other substances. This comorbidity is even more pronounced in clinical populations, particularly among homeless groups [6] and in acute psychiatric wards [7,8] where patients with schizophrenia are particularly frequent. The prevalence of SUD varies considerably between studies, i.e. 24-50% [7-9]. This is explainable by differences in substance use levels in the catchment areas and by intake policies. Another explanation could be insufficient diagnostic practice [10]. Several studies have found under-diagnosis of SUD in psychiatric hospitals [11,12]. There is also evidence that this group of patients do not receive health services according to their needs. Harris and Edlund found that mental health programs provided substance use services to only 31% of the clients evidencing severe mental illness with SUD [13].

These investigations have laid a sound basis for the knowledge on comorbidity among inpatient populations. Little, however, is known about the prevalence of SUD in Community Mental Health Centres (CMHCs), where the clinical population differs from that of an acute psychiatric ward by having predominantly anxiety disorders and non-psychotic affective disorders. Further, it is insufficiently studied whether patients with substance use disorders differ from patients without such comorbidity and whether the CMHCs differentiate their treatment accordingly.

In a recent study of CMHCs in Norway, we found that only 10% of the patients had received ICD-10 diagnoses [14] of SUD [15]. The obvious explanation was under-detection by the clinicians. In addition to the ICD-10 diagnoses, the centres used the Health of the Nation Outcome Scales (HoNOS) which includes a score on substance use problems, the Alcohol Use Scale (AUS) and the Drug Use Scale (DUS). There were some discrepancies between these measures regarding which patients were scored as having a clinical problematic use of alcohol and/or drugs. By combining these measures, however, we were able to identify all patients that were scored as having a clinically significant use of alcohol and/or other substances or diagnosed with a substance use disorder. These patients were defined as the SUD group. Patients that received a low score, indicating no clinical problem, on the substance use measures and did not receive an ICD-10 diagnosis of SUD were defined as the No SUD group. Hence, the grouping variable consists of the HoNOS, the AUS and the DUS, all validated measures, together with the ICD-10 diagnoses, which were based on routine clinical assessments.

In this paper we compare the SUD group with the No SUD group

The aims of this paper are to compare the patients in CMHCs with and without SUD in regard to 1) differences in socio-demographic characteristics, 2) the level of morbidity, 3) the prevalence of different diagnostic categories, 4) differences in health services provided and 5) differences in the level of improvement in psychiatric symptoms.

## Methods

### Study design

This is a cross-sectional study based upon data from the evaluation of the National Plan for Mental Health [16,17]. In the original study, eight Community Mental Health Centres (CMHCs) in both rural and urban areas of Norway were investigated during three separate 4 week periods in 2002, 2005 and 2007. Their total catchment area was about 450 000 inhabitants, i.e. about 10% of the Norwegian population. Specific forms were completed by clinicians, general practitioners, patients and their relatives. For this paper, we have focused on the clinician assessment forms from 2007.

The clinicians at the CMHCs, i.e. psychiatrists, psychologists, psychiatric nurses and clinical social workers, were asked to complete standardized forms for all inpatients and outpatients seen within the four week period. A total of 2154 patients were included. This was about half of all patients reported to the National Patient Register (NPR) from these CMHCs in a similar 4-week period a few months later, as NPR data from the study period was unavailable. We had no information on eligible patients not included in the study. Details on the methodology are described elsewhere [15].

### Research instruments

Demographic, administrative and clinical information was recorded for each patient. One primary and two secondary ICD-10 diagnoses [14] were recorded on the basis of routine clinical assessments. No structured clinical interview was used. The Health of the Nations Outcome Scales (HoNOS) [18,19] were used to measure severity of psychiatric problems in 12 areas including substance use. The scale ranges from "no problem" (score 0) through "mild but clinically significant problem" (score 2) to "severe problem" (score 4). The Alcohol Use Scale (AUS) and the Drug Use Scale (DUS) [20,21] were used to rate the severity of alcohol and drug use, respectively. These are 5 point scales ranging from "abstinence" (score 1) through "abuse" (score 3) to "addiction with hospitalization" (score 5). Before the first two surveys in 2002 and 2005, the clinicians at the eight CMHCs were trained in rating the HoNOS, the AUS and the DUS. Before each of the three surveys they had an optional practice on case vignettes. In 2002, intraclass correlation coefficients (ICC) for the HoNOS were calculated and ranged from 0.60 to 0.89 for the subscales (T. Ruud, personal communication). These coefficients were considered acceptable [22,23]. We found the same prevalence rates of SUD measured by the ICD-10 diagnoses, the HoNOS, the AUS and the DUS between 2002 and 2007, hence we concluded there was consistency in how these instruments were used between these years [15].

#### Substance use variables

We defined the *SUD group *as having fulfilled one or more of the following criteria: 1) a diagnosis of a substance related disorder, ICD-10 F10-19, as first, second or third diagnosis, 2) a high degree of alcohol and/or drug use measured by the HoNOS item three, scored 2-4, 3) a high degree of alcohol use measured by the AUS, scored 3-5, or 4) a high degree of drug use measured by the DUS, scored 3-5. The *No SUD group *was defined by not fulfilling any of the above criteria.

#### Socio-demographic variables

The socio-demographic variables consisted of *age, gender, paid work *(yes/no), *in relationship *(yes/no), *living alone *(yes/no) and *ethnicity *(Norwegian / non-Norwegian). *No paid work *was defined as "in education", "working at home", "on rehabilitation benefit", "on disability benefit", "on retirement pension" and "other". *In relationship *was defined as "married" or "cohabitant", while *not in relationship *was defined as "unmarried", "widowed", "separated" or "divorced".

#### Variables regarding the level of morbidity

The variables about the patients' level of morbidity were the *HoNOS *(except the substance use item). The substance use item of the *HoNOS *was used as one of the SUD group defining criteria. We categorized the *HoNOS *scores into two groups: 1) no clinically relevant problem (scores 0-1) and 2) clinically relevant problem (scores 2-4).

#### Variables regarding the prevalence of different diagnostic categories

The ICD-10 diagnoses were grouped into *psychotic disorders *(F20-29), *mood disorders *(F30-39), *anxiety disorders *(F40-49), *personality disorders *(F60-69) and *other psychiatric disorders *(F50-59, F70-99)

#### Variables regarding the health services provided

The variables about the health services provided consisted of *psychiatric healthcare received during the last 12 months *and *additional questions about the services in total*. The original 6 items of *psychiatric healthcare received during the last 12 months *were categorized into 3 groups; "outpatient or day service at CMHC", "inpatient service at CMHC or hospital" and "outpatient or inpatient addiction treatment". They were scored as "no", "0-6 months", "7-11 months" and "all the time". These categories were dichotomized into "no" and "0-12 months" for the analyses. The *additional questions about the services in total *were "is the patient treated at the right competency level" (scored as "unnecessarily high", "right" or "too low"), "are the services sufficiently comprehensive", "are the most important needs of the patient met", "are several services cooperating in making an "Individual Plan" for the patient", and "is the patient also treated in a psychiatric hospital". These questions were scored as "yes / no / I don't know/ not applicable". The latter two options were taken out of the analysis. An "Individual Plan" means a tailored, comprehensive treatment plan that all patients with a chronic disease are entitled to have according to Norwegian law.

#### Variables regarding the level of improvement from psychiatric symptoms

The variables were scored on a 7-point scale (1: much worse, 2: a bit worse, 3: no change, 4: a little change, 5: better, 6: much better, 7: a lot better). These items were grouped into two groups: 1) worse / no change (scores 1-3) and 2) better (scores 4-7). This scale is based on the clinicians' subjective evaluation of the patients' improvement on the day of the survey, regardless of their length of treatment.

### Statistics

When comparing two groups the Student's t-test was used for continuous variables and the Pearson's chi-square test was used for categorical variables. We performed logistic regression analyses to select the adjustment variables. An α-level of 0.05 was chosen when deciding which adjustment variables to include. We included the following variables for the adjusted analyses; *age, gender, in relationship, overactive, aggressive, disruptive or agitated behaviour (honos item 1), non-accidental self-injury (honos item 2) *and *problems with activities of daily living (honos item 10)*. We also adjusted for the interaction between *age *and *problems with activities of daily living*. The explanatory variable "problems with relationships" (honos item 9) was not adjusted for *in relationship*, as this was not clinically meaningful. For the main analyses we did Bonferroni correction of the alfa-level due to the number of tests to avoid Type I statistical error. When an explanatory variable lost its significance due to the adjusted analyses, we ran a series of regression analyses with each adjustment variable to examine which one resulted in the greatest change in the beta coefficient of the explanatory variable. Finnally, we performed Generalized Estimating Equations analyses (GEE) with exchangeable working correlation and robust variance estimation. This was done to adjust for nesting within the CMHCs in the adjusted analyses. The GEE analyses only gave minor changes in the results compared to the regression analyses. Because *gender *was shown to be an important adjustment factor, we did stratified analyses by gender. As these analyses only showed small differences in the odds ratios between men and women, this issue is not elaborated any further. The analyses were performed using SPSS version 18.0 [24].

### Ethics and consent issues

The study was approved by the Norwegian Data Inspectorate and the Norwegian Regional Ethics Committee.

## Results

Table 1 shows the background variables of the non-substance use disorder group, n = 1786, and the substance use disorder group, n = 368. The mean age amongst the patients in total was 39.3 years with no statistically significant difference between the two groups. The patients in the SUD group were more often male, less often in a relationship and more often living alone compared to the No SUD group. Even though these differences were statistically significant, the effect-sizes were quite small [25]. There were no differences in ethnicity between the two groups.

**Table 1:** Background variables of the non substance use disorder (No SUD) and substance use disorder (SUD) groups

Background variables	**No SUD**^**a**^ N = 1786	**SUD**^**a**^ N = 368	Effect-sizes^b^	p-value^c^
Age	39.4 (12.9)	38.7 (12.9)	0.058 (0.029)	0.312
Male gender	601 (34.9)	196 (55.1)	0.156	< 0.001
Not in relationship	987 (55.9)	266 (73.1)	0.131	< 0.001
Living alone	553 (34.4)	166 (49.6)	0.119	< 0.001
No paid work	1296 (73.5)	287 (79.3)	0.050	0.021
Norwegian	1638 (92.3)	348 (94.8)	-0.037	0.088

a) Age is presented as mean (SD) all other variables are presented as n (%). Valid percentages are given.

b) Cohen's *d *(effect size *r*) is used for age, for all other variables the phi-coefficient is used

c) The student's T-test is used for age, for all other variables the χ^2^-test is used.

We examined if the severity of morbidity as measured by the HoNOS predicted being a SUD patient (table 2). We found that the SUD group had significantly more problems with "overactive, aggressive, disruptive or agitated behaviour", "non-accidental self-injury", "problems with relationships", "problems with activities of daily living" and "problems with occupation and activities". These results were not altered when other variables were adjusted for. The SUD patients also seemed to have more "cognitive problems" and "problems with living conditions". These results, however, changed to a non-significant level after adjustment and we found that *gender *had the greatest impact in the adjusted analyses.

**Table 2:** Prevalence rates, multivariate unadjusted and adjusted odds ratios (OR) with 99.9% confidence intervals (CI) indicating the odds for being a patient with substance use disorder (SUD) rather than being a patient without SUD in regard to the level of morbidity measured by the Health of the Nation Outcome Scales

	NO SUD^a^ N = 1786	SUD^a^ N = 368	Odds ratio			
			Unadjusted	99.9% CI	Adjusted^b^	99.9% CI
*Health of the Nation Outcome Scale* *(scores 2-4) *Score 0-1 is reference OR = 1						
01 - Overactive, aggressive, disruptive or agitated behaviour	131 (7.5)	64 (17.7)	**2.7**	1.6-4.5	**2.3**	1. 3-4.0
02 - Non-accidental self-injury	146 (8.3)	57 (15.7)	**2.1**	1.2-3.5	**2.1**	1.2-3.8
04 - Cognitive problems	261 (14.9)	79 (22.1)	**1.6**	1.0-2.6	1.4	0.8-2.2
05 - Physical illness or disability problems	405 (23.0)	95 (26.4)	1.2	0.8-1.8		
06 - Problems associated with hallucinations and delusions	205 (11.6)	46 (12.8)	1.1	0.6-2.0		
07 - Problems with depressed mood	983 (55.9)	213 (59.2)	1.1	0.8-1.7		
08 - Other mental and behavioural problems	988 (73.0)	207 (73.7)	1.0	0.6-1.7		
09 - Problems with relationships	974 (55.3)	245 (67.3)	**1.7**	1.1-2.5	**1.6**	1.1-2.5
10 - Problems with activities of daily living	517 (29.3)	159 (43.9)	**1.9**	1.3-2.8	**1.6**	1.1-2.4
11 - Problems with living conditions	120 (6.8)	54 (14.9)	**2.4**	1.4-4.2	1.6	0.9-3.1
12 - Problems with occupation and activities	493 (28.1)	149 (41.3)	**1.8**	1.2-2.6	**1.6**	1.1-2.4

Odds ratios with p-values < 0.0013 are given in bold face

^a ^The variables are presented as n (%). Valid percentages are given.

^b ^Generalized Estimating Equations are used. All variables are adjusted for *age, gender *and *in relationship *except "problems with relationships" which is adjusted for *age *and *gender.*

**Table 3:** Prevalence rates, multivariate unadjusted and adjusted odds ratios (OR) with 99.9% confidence intervals (CI) indicating the odds for being a patient with substance use disorder (SUD) rather than being a patient without SUD in regard to type of psychiatric diagnosis received

*Having received a psychiatric diagnosis* Not having the disorder is reference OR = 1	No SUD^a^ N = 1786	SUD^a^ N = 368	Odds ratios			
			Unadjusted	99.9% CI	Adjusted^b^	99.9% CI
Psychotic disorder	243 (13.8)	39 (10.7)	0.8	0.4-1.4		
Mood disorder	817 (46.5)	123 (33.9)	**0.6**	0.4-0.9	**0.7**	0.4-1.0
Anxiety disorder	684 (38.9)	83 (22.9)	**0.5**	0.3-0.7	**0.5**	0.3-0.8
Personality disorder	294 (16.7)	53 (14.6)	0.9	0.5-1.4		
Other psychiatric disorder	233 (13.3)	41 (11.3)	0.8	0.5-1.5		

Odds ratios with p-values < 0.0013 are given in boldface

^a^The variables are presented as n (%). Valid percentages are given.

^b ^Generalized Estimating Equations are used. All variables are adjusted for *age, gender *and *in relationship*

In this sample only 36.1% of the patients received more than one diagnosis [15] which would make it difficult to look at the prevalence of psychiatric disorders beside SUD. However, our definition of SUD is based upon the HoNOS, the AUS and the DUS besides the ICD-10 diagnoses. Consequently, we can look at comorbidity between SUD and other illnesses. In the SUD group, 268 patients (72.8%) received diagnoses of somatic or psychiatric disorders other than SUD. Having a mood disorder and having an anxiety disorder was more common amongst No SUD patients and these results were not altered after adjustment.

We examined if receiving certain health services predicted being a SUD patient (table 4). We found that receiving outpatient or day service at the CMHC was less common amongst the SUD patients. This result was not altered when adjusted for other variables. Receiving inpatient service at the CMHC or hospital was more common among the SUD patients, and even though the effect-size (OR) was not altered by more than 0.3 units, the result was no longer statistically significant after adjustment. We found that *in relationship *had the greatest impact in the adjusted analyses. Receiving outpatient or inpatient addiction treatment was, not surprisingly, more common among the SUD patients and this was not altered after adjustment. The SUD patients were more often reported to be treated at too low a competency level, and controlling for other variables did not alter this result. The therapists did not perceive the SUD patient's services to be sufficiently comprehensive. This result, however, was altered to a non-significant level when adjusted for, even if the change in effect size was only 0.2 units. We found that *overactive, aggressive, disruptive or agitated behaviour *(honos item 1) and *problems with activities of daily living *(honos item 10) had the greatest impact in the adjusted analyses. Having the patient's most important needs met was less common among the SUD patients, but this result also became non-significant after adjustment even if the effect size was only changed by 0.2 units. *Overactive, aggressive, disruptive or agitated behaviour *(honos item 1) and *problems with activities of daily living *(honos item 10) had the greatest impact in the adjusted analyses. Having several services cooperating in making an individual plan for the patient was more common amongst the SUD patients, but the result became non-significant after adjustment. *Gender *and *in relationship *had the greatest impact in the adjusted analyses.

**Table 4:** Prevalence rates, multivariate unadjusted and adjusted odds ratios (OR) with 99.9% confidence intervals (CI) indicating the odds for being a patient with substance use disorder (SUD) rather than being a patient without SUD in regard to type of health services received

	No SUD^a^ N = 1786	SUD^a^ N = 368	Odds ratios			
			Unadjusted	99.9% CI	Adjusted^b^	99.9% CI
*Type of psychiatric healthcare received during the last 12 months* No treatment received during the last 12 months is ref. OR = 1						
Outpatient or day service at CMHC	1654 (94.4)	259 (77.3)	**0.2**	0.1-0.3	**0.2**	0.1-0.4
Inpatient service at CMHC or hospital	372 (25.8)	115 (38.1)	**1.8**	1.2-2.7	1.5	0.9-2.4
Outpatient or inpatient Addiction treatment	23 (1.7)	148 (46.7)	**51.5**	23.9-110.9	**55.8**	23.8-130.9
*Additional questions about the services in total*						
The patient is treated at too low a level Too high a level or sufficient level is reference OR = 1	45 (2.6)	34 (9.4)	**4.0**	1.9-8.4	**2.8**	1.1-6.7
The services are sufficiently comprehensive Not comprehensive is reference OR = 1	1180 (87.3)	218 (76.8)	**0.5**	0.3-0.8	0.7	0.4-1.4
The patient's most important needs are met Important needs are not met is reference OR = 1	1460 (91.5)	267 (82.2)	**0.4**	0.2-0.7	0.6	0.3-1.1
Several services are cooperating in making an individual plan for the patient Not cooperating is reference OR = 1	326 (39.6)	112 (52.1)	**1.7**	1.0-2.7	1.3	0.7-2.2
The patient is also treated in a psychiatric hospital Not treated in psych. hospital is reference OR = 1	81 (14.3)	36 (23.1)	1.8	0.9-3.7		

Odds ratios with p-values < 0.0013 are given in boldface

^a^The variables are presented as n (%). Valid percentages are given.

^b ^Generalized Estimating Equations are used. All variables are adjusted for *age, gender, in relationship, Overactive, aggressive, disruptive or agitated behaviour *(honos item 1), *Non-accidental self-injury *(honos item 2), *Problems with activities of daily living *(honos item 10) and the interaction term age* problems with activities of daily living

We examined if the degree of recovery predicted being a SUD patient. We found that having no change or getting worse in regard to "psychological problems" was more common among SUD patients and this result was not altered when adjusted for other variables (table 5). Having "psychiatric problems", "problems with social functioning" and "problems with practical functioning" was also more common amongst SUD patients, but changed to a borderline significant level after being adjusted with a change in effect sizes between 0.1-0.3 units. Having no change or getting worse in regard to "problems with close relations" was more common amongst the SUD patients, but changed to a non significant level after being adjusted, with a change in effect size of only 0.3 units. We found that *gender *was the adjustment factor that was of greatest importance in changing the significance level of all the explanatory factors. For the explanatory factors "psychiatric problems", "problems with close relations" and "problems with social functioning" the adjustment factor *in relationship *was also of importance.

**Table 5:** Prevalence rates, multivariate unadjusted and adjusted odds ratios (OR) with 99.9% confidence intervals (CI) indicating the odds for being a patient with substance use disorder (SUD) rather than being a patient without SUD in regard to recovery

*No change or getting worse from:* Getting better is reference OR = 1	No SUD^a^ N = 1786	SUD^a^ N = 368		Odds ratios		
			Unadjusted	99.9% CI	Adjusted^b^	99.9% CI
Psychiatric symptoms	516 (29.3)	155 (42.7)	**1.8**	1.2-2.6	1.5	1.0-2.2
Psychological symptoms	576 (32.8)	170 (47.0)	**1.8**	1.2-2.6	**1.5**	1.0-2.3
Problems with close relations	863 (49.4)	213 (59.0)	**1.5**	1.0-2.1	1.2	0.8-1.9
Problems with social functioning	702 (40.0)	189 (51.9)	**1.6**	1.1-2.3	1.5	1.0-2.2
Problems with practical functioning	800 (46.0)	212 (58.9)	**1.7**	1.1-2.5	1.5	1.0-2.2
Problems with working disability	1050 (60.6)	243 (67.1)	1.3	0.9-1.9		
Behavioural problems	1069 (65.9)	243 (70.4)	1.2	0.8-1.9		

Odds ratios with p-values < 0.0013 are given in boldface

^a^The variables are presented as n (%). Valid percentages are given.

^b ^Generalized Estimating Equations are used. All variables are adjusted for *age, gender, in relationship, Overactive, aggressive, disruptive or agitated behaviour *(honos item 1), *Non-accidental self-injury *(honos item 2), *Problems with activities of daily living *(honos item 10) and the interaction term age* problems with activities of daily living

## Discussion

In this study we investigated the treatment of patients with SUD in Community Mental Health Centres (CMHCs) which typically treat patients with non-psychotic disorders such as anxiety disorders, non-psychotic affective disorders, adjustment difficulties and personality problems. The main findings are that, also in outpatient settings, the SUD group differs from the No SUD group in several ways not often systematically met with adequate treatment approaches.

Our first findings are that the patients in the SUD group are more often male, less often in a relationship and more often living alone. This is largely consistent with other epidemiological and clinical studies [4,5,26-28], except for one study where only the gender difference was found [29]. These findings indicate that people with SUD are a more vulnerable group. Consequently, therapists should plan treatment accordingly.

The second finding is that the SUD group has more severe morbidity as measured by the HoNOS with higher scores on five out of eleven parameters when adjusted for *age, gender *and *in relationship*. This means that the SUD group has more problems with aggressive behaviour, self harm, relationships, occupation and activities of daily living. It is therefore essential that these problems are targeted in their treatment plan. We found one other study targeting CMHCs with comparable measures [30]. In this study of patients with schizophrenia in inpatient and outpatient clinics, the misuse group was found to have higher sum scores on the HoNOS compared to the non-misuse group. In addition, Fowler et al found higher mean scores on the Symptom Check List-90 revised (SCL-90R) [31-33] on all sub-scores amongst the SUD group compared to the No SUD group amongst patients with schizophrenia [34].

The third finding is that the SUD patients have lower prevalences of anxiety and depression compared to patients without SUD. This finding is in contrast to most studies in the field indicating a higher prevalence of most comorbid disorders in SUD patients[1,35,36]. However, Bonsack et al found a similar pattern for anxiety and depression amongst patients in an acute psychiatric ward, but with higher prevalences for other psychiatric diagnoses amongst the SUD group compared to the No SUD group [28]. Our finding could have several explanations. Firstly, the different CMHCs may have recruited different numbers of SUD and No SUD patients due to different catchment areas. Secondly, it could be a case of competing risks; patients with SUD need less other morbidity for referral to CMHCs; thus not reflecting the comorbidity in the general population. Thirdly, less severe mental illnesses, like anxiety and depression, with comorbid SUD may be more often referred to substance use treatment centres. This could be detrimental to outcome, as patients treated in substance abuse treatment facilities may not receive adequate attention for their comorbid psychiatric illness. Bakken et al did a six year follow-up study of substance abusers in inpatient and outpatient addiction treatment facilities. They found that the number and specific types of axis 1 and axis 2 disorders with the level of SUD at admission were all independent predictors of a high level of mental distress at follow-up [37]. This underlines the need for good diagnostic and screening routines along with the competence to treat this comorbidity in an integrated treatment program.

The fourth and important finding is that patients with SUD in these CMHCs are treated differently. The patients in the SUD group receive less outpatient treatment compared to the No SUD group. In addition, the clinicians rate the patients in the SUD group as being treated at too low a competency level. One possible explanation might be that the therapists feel they lack the competence or the resources to treat these patients in an outpatient setting. In a phenomenological study of clinicians in mental health centres Deans et al found that the clinicians felt unprepared and that they were lacking knowledge of dual diagnosis patients [38]. This is in accordance with other studies that describe difficulties in implementing new knowledge and guidelines regarding comorbid patients in mental health care [39,40]. This highlights the need for establishing and implementing good treatment strategies for this group of patients.

Our final finding is that the patients in the SUD group have poorer outcomes in regard to recovery from psychological problems. In addition, they have poorer outcomes on three out of the remaining six items at a borderline significant level after adjustment for other variables. This is in accordance with previous findings of poorer treatment outcomes for patients with co-occurring disorders [41] and is in line with the other findings of this paper, that is, that these patients have greater morbidity and receive less adequate help for their problems.

There are several limitations to this study. This study is part of an evaluation of the National Plan for Mental Health that was adapted to the study aims, the prevalence of SUD was measured by a composite adapted approach, and there was no structured clinical interview used to assess diagnoses. Further, the variables regarding the level of improvement from psychiatric symptoms were based on the clinicians' subjective evaluation of the patients' improvement, regardless if the clinician knew the patient for a longer or shorter period of time. Prior to the surveys in 2002 and 2005 the clinicians were trained in using the HoNOS, the AUS and the DUS while they only had optional training on case vignettes prior to the survey in 2007. However, comparing the results from 2007 with the results from 2002 in regard to the prevalence of SUD measured on the ICD-10 F10-19 diagnoses, the HoNOS, the AUS and the DUS, we found no significant differences [15]. For further studies we would recommend to include screening procedures for SUD, i.e. the Alcohol Use Identification Test (AUDIT) [42] and the Drug Use Identification Test (DUDIT) [43], and valid diagnostic procedures, such as the Structured Clinical Interview for DSM-IV (SCID) [44] or the Psychiatric Research Interview for Severe Mental disorders (PRISM) [45]. One might also include measures like the HoNOS, the Symptom Check List (SCL-90R) or the Addiction Severity Index (ASI) [46] to enable basic comparisons between studies. Finally, the representativeness of outpatient clinics in Norway might be questioned, both according to the prevalence and type of substance use in the catchment areas and the clinical routines in the units. It is obviously important to confirm these findings in further studies. However, the findings underline the need for targeted treatment approaches for patients comorbid with SUD in psychiatric outpatient units.

## Conclusion

Patients with SUD in CMHCs are more frequently male, single and living alone, have a higher level of morbidity, less anxiety and mood disorders, less outpatient treatment and have less improvement in regard to recovery from psychological symptoms compared to patients with no SUD. CMHCs need to implement systematic screening and diagnostic procedures in order to detect the special needs of substance abusing patients and improve their treatment.

## Competing interests

The authors declare that they have no competing interests.

## Authors' contributions

All the authors fulfil the Vancouver requirements for authorship. TR and RG have been involved in the conception, design and acquisition of the data. HW and LW have been involved in analysing and interpreting the data. LW has drafted the manuscript. All authors have been involved in revising the manuscript critically for important intellectual content and approved the version to be published.

## Authors' information

LW is a psychiatrist and PhD-fellow at the Norwegian Centre for Addiction Research at the Institute of Clinical Medicine, University of Oslo. HW is a psychiatrist and professor at the Norwegian Centre for Addiction Research at the Institute of Clinical Medicine, University of Oslo. TR is a psychiatrist, professor at the Institute of Clinical Medicine, University of Oslo, and Head of the Department of Research and Development at the Division of Mental Health Services, Akershus University Hospital. RG is a psychologist, Head of the Department of Research and Development at the Alcohol and Drug Treatment Health Trust in Central Norway and Associate professor at the Norwegian Centre for Addiction Research at the Institute of Clinical Medicine, University of Oslo.

## Pre-publication history

The pre-publication history for this paper can be accessed here:

http://www.biomedcentral.com/1471-244X/11/93/prepub
